# Simple technology for recycling phosphate from wastewater to farmland in rural areas

**DOI:** 10.1007/s13280-017-0976-9

**Published:** 2017-11-21

**Authors:** Hisao Ohtake, Kenji Okano, Masashi Kunisada, Hiroyuki Takano, Masaya Toda

**Affiliations:** 1 Phosphorus Atlas Research Institute, Waseda University, Wakamatsu-cho 2-2, Shinjuku-ku, Tokyo, 162-0056 Japan; 2 Department of Biotechnology, Graduate School of Engineering, Osaka University, 2-1 Yamada-oka, Suita, Osaka 565-0871 Japan; 3 Mikuni Pharmaceutical Industrial Co., Ltd., 2-35 Kamisu-cho, Toyonaka, Osaka 561-0823 Japan; 4 Research & Development Center, Taiheiyo Cement Co., 2-4-2 Osaku, Sakura, Chiba 285-8655 Japan; 5 Research & Development Laboratory, Onoda Chemical Industry Co., Ltd., 39-13 Miyamoto-cho, Itabashi-ku, Tokyo 174-0054 Japan

**Keywords:** Amorphous calcium silicate hydrates, Bifunctional agent, Mobile plant, Phosphate recovery, Wastewater treatment plant

## Abstract

A simple technology for phosphate (P_*i*_) recovery has been developed using a bifunctional adsorption–aggregation agent. The bifunctional agent was prepared by soaking calcium silicates in hydrochloric acid solution. Importantly, recyclable calcium silicates were available almost free of charge from the cement industry and also from the steel industry. The acid treatment was essential not only for enhancing the ability of calcium silicates to remove P_*i*_ from aqueous solution but also for enabling the high settleability of removed P_*i*_. On-site experiments using a mobile plant showed that approximately 80% P_*i*_ could be recovered from anaerobic sludge digestion liquor at a wastewater treatment plant. This technology has the potential to offer a simple, compact service for recycling P_*i*_ from wastewater to farmland in rural areas.

## Introduction

Various technologies are potentially applicable to phosphate (P_*i*_) recovery from secondary phosphorus (P) resources such as sewage sludge, incinerated sludge ash, animal manure, and steelmaking slag (Ohtake and Okano 2015). However, their implementation has often been economically infeasible because of the high cost of plant development, construction, and operation. In particular, P_*i*_ recovery in small wastewater treatment plants (WWTPs) has been virtually untouched mainly due to cost and labor shortage problems. There are approximately 2100 sewage treatment plants currently operating in Japan. More than 70% of these plants have the capacity to treat wastewater of less than 5000 t d^−1^ (approximately 20 000 population equivalents (p.e.)) (Okano et al. 2016a). In addition, approximately 1000 small plants are operating to treat blackwater and septic tank sludge in rural areas where no sewage service is available. Since these plants are mostly operated by less than 10 workers, it is hard to embark on P_*i*_ recovery which has been considered as an extra service in the wastewater treatment sector. P_*i*_ recovery in small WWTPs is likely a common challenge for recycling nutrients from wastewater to farmland in rural areas.

Crystalline calcium silicate hydrates (CSHs) are formed in hyperalkaline, hydrothermal environments (Chen et al. 2009). Crystalline CSHs have a wide range of structures with various Ca/Si molar ratios (Shaw et al. 2000). However, only crystalline CSHs with a low Ca/Si molar ratio of 1.0–1.2, such as tobermorite (Ca_5_Si_6_O_16_(OH)_2_·4H_2_O) (Berg et al. 2005) and xonotlite (Ca_6_Si_6_O_17_(OH)_2_) (Chen et al. 2009), have been used for P_*i*_ removal from wastewater. Namely, they have been used as a seed for hydroxyapatite crystallization to remove P_*i*_ from wastewater. However, hydroxyapatite crystallization requires long reaction time in a complicated reactor for P_*i*_ removal. Amorphous calcium silicate hydrates (A-CSHs), which had a high Ca/Si molar ratio of 2.0 or greater, could be chemically synthesized using unlimitedly available, inexpensive materials such as siliceous shale and Ca(OH)_2_ (Okano et al. 2013). Importantly, the chemically synthesized A-CSHs could serve not only as a P_*i*_ adsorbent but also as an aggregation agent in aqueous solution (Okano et al. 2015). P_*i*_ removed by A-CSHs exhibited better settleability, filterability, and dewaterability than P_*i*_ precipitated with conventional CaCl_2_ and Ca(OH)_2_. Moreover, unlike CaCl_2_ and Ca(OH)_2_, no significant carbonate inhibition was observed with P_*i*_ removal by A-CSHs.

Recyclable calcium silicates are available almost free of charge from the cement industry and also from the steel industry. For example, concrete sludge (CS) is an alkaline waste slurry containing CSHs as a major component. CS is abundantly available at construction sites where more concrete materials are generated than required (Okano et al. 2016b). Conventionally, CS has been disposed of as a landfill material after solid–liquid separation followed by neutralization with a strong acid (Tsunashima et al. 2012). However, since this is costly and wasteful, it is desirable to develop an alternative technology option that can valorize unwanted CS for the cement and construction industry. On the other hand, steelmaking slag is the most abundantly available byproduct, which contains calcium silicates as a major component, in the steel industry (Matsubae et al. 2015). Since the amount of steelmaking slag produced by a steelmaking process is very large, it is a matter of great concern for the steel industry to effectively recycle this byproduct.

Previously, we have shown that a substitute for chemically synthesized A-CSHs could be obtained simply by soaking CS in hydrochloric acid solution for a short period of time (Okano et al. 2016b). Like chemically synthesized A-CSHs, the acid-treated CS could serve as a bifunctional adsorption–aggregation agent for P_*i*_. Since recyclable calcium silicates such as CS and steelmaking slag are available almost free of charge, this finding may pave the way for the development of a simple, low-cost technology for P_*i*_ recovery in small WWTPs. In the present study, we compared the potential of chemically synthesized A-CSHs and acid-treated CS for P_*i*_ recovery in small WWTPs using a mobile pilot-scale plant. Based on the results of on-site experiments, we discussed the possibility to offer new technology options, including an on-site P_*i*_ recovery service, to small WWTPs for recycling P_*i*_ from wastewater to farmland in rural areas.

## Materials and methods

### Sample preparation

A-CSHs, which had a high Ca/Si molar ratio of 2.0 or greater, were chemically synthesized using siliceous shale (M-rite) and Ca(OH)_2_ as described previously (Okano et al. 2013). M-rite was abundantly available from the cement industry. The SiO_2_ content of M-rite was approximately 76% (w/w). Autoclaved lightweight concrete (ALC) particles, a byproduct of the building material industry, were employed as control crystalline CSHs (Liu et al. 2001). The ALC particles contained crystalline tobermorite and quartz as principal components. ALC particles were sieved through a 100-μm-mesh-size stainless steel screen before use.

Unused CS was taken from a ready-mix concrete plant, washed with water, and dewatered using a filter press (AK175, Kyokuto Sangyo Co., Ltd., Tokyo, Japan). The filter cake was subjected to air drying at room temperature and ground using a mortar and pestle to obtain CS particles. Then CS particles were soaked in 1.3 M HCl at a concentration of 0.1 g mL^−1^ for 60 min to make acid-treated CS a substitute for chemically synthesized A-CSHs. During the HCl-soaking treatment, the mixture was continuously stirred by a magnetic stirrer at 600 rpm at room temperature. When required, the acid-treated CS slurry was separated into solid and liquid fractions by centrifugation at 2000×*g* at room temperature for 10 min. To study the effect of the surface electric charge of particles on P_*i*_ settleability, 5 mL acid-treated CS slurry was mixed with 100 mL of 1 M NaCl. The mixture was stirred by a magnetic stirrer at 200 rpm at room temperature for 30 min. Then, solids were recovered from the mixture by centrifugation at 2000 g at room temperature for 10 min before being used for P_*i*_ settling experiments.

### Characterization

Electron microscopy was performed at 200 kV using an H-800 transmission electron microscopy system (Hitachi Ltd., Tokyo, Japan) and at 1.5 kV using a JSM-7600F scanning electron microscopy (SEM) system (JEOL Ltd., Tokyo, Japan). For SEM analysis, samples were dried overnight under vacuum. Powder X-ray diffraction analysis was carried out using a D8 ADVANCE diffractometer (Bruker AXS K. K., Japan). For X-ray diffraction analysis, samples were dried by heating at 100 °C for 10 h. The particle size distribution was determined using a Microtrac particle size analyzer (model 9320-X100, Nikkiso Co., Ltd., Tokyo, Japan). The BET specific surface area was measured from N_2_ adsorption isotherms using an ASAP-2400 adsorption analyzer (Micrometrics Inc., Norcross, GA, USA). The chemical composition of CS particles before and after acid treatment was determined using an X-ray fluorescence (XRF) spectrometer (ZSX100e, Rigaku Co., Tokyo, Japan). Heavy metals, including Cd, Pb, Ni, and Cr, were determined using a polarized Zeeman atom absorption spectrophotometer (model Z-5300, Hitachi High-Technologies Co., Tokyo, Japan).

### P_*i*_ recovery test

Preliminary experiments were carried out in laboratory to compare the P_*i*_ removability, settleability, and filterability of chemically synthesized A-CSHs, ALC, CS, and acid-treated CS. For laboratory experiments, a synthetic anaerobic sludge digestion liquor, designated test solution, was prepared by dissolving 392 mg of KH_2_PO_4_, 1.89 g of NH_4_Cl, and 3.36 g of NaHCO_3_ in 1 L of deionized water. The test solution was designed to have essentially the same P_*i*_ concentration (272 mg P_*i*_ L^−1^) and buffering capacity as a typical anaerobic sludge digestion liquor. P_*i*_ recovery experiments were carried out by adding adsorbents to 200 mL of test solution at varied concentrations. The mixture was stirred at 250 rpm at room temperature. Samples were taken from the mixture at various time intervals and filtered through a 0.22-μm-pore-size Millex-GV filter (Millipore). The filtrate P_*i*_ was determined by the molybdenum blue method described by Murphy and Riley (1962). Total P was determined as P_*i*_ after autoclaving a sample at 121 °C for 30 min (APHA, AWWA, and WEF 2012). Citrate-soluble P (C-P_2_O_5_) in recovered products was determined as P_*i*_ solubilized by 2% citric acid (Braithwaite 1987). After 20 min of P_*i*_ removal, the reaction mixture was transferred to a 200-mL graduated cylinder (4.0 cm in diameter) to assess the settleability of recovered P. After 10 min of free sedimentation, 180 mL (90% of the original volume) of the supernatant was gently removed using a siphon. Settleability was evaluated by determining the percentage of total P that remained in the cylinder after siphoning the supernatant.

### Mobile plant

**Fig. 1:**
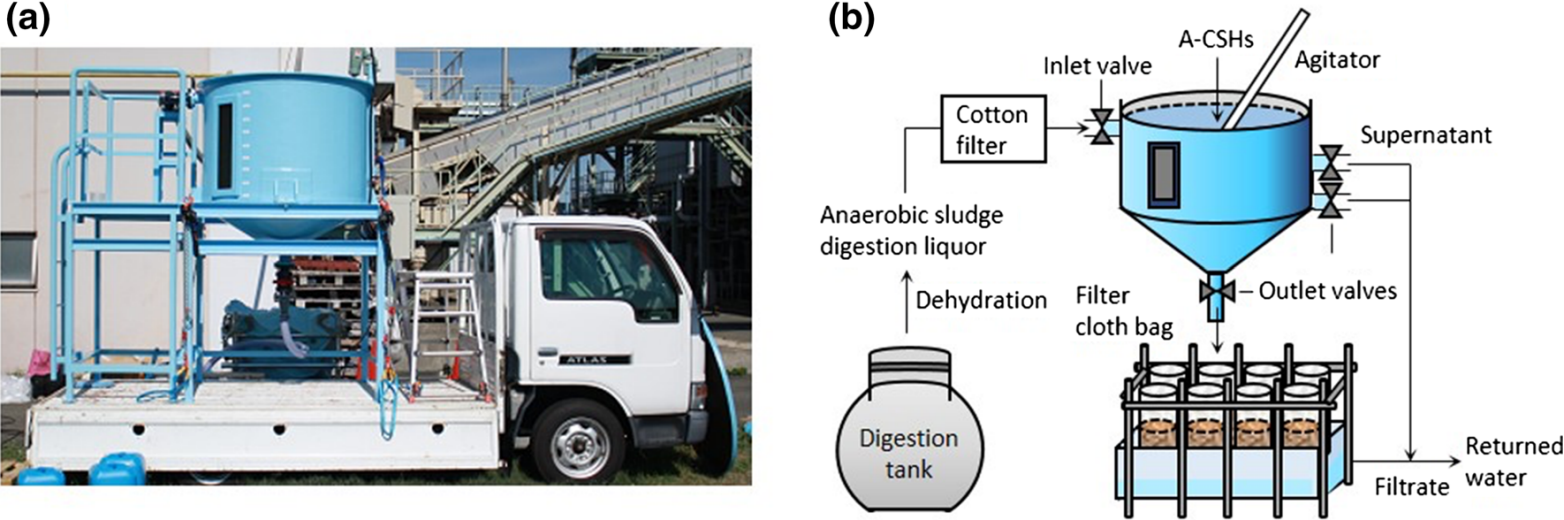
Mobile plant (**a**) and the on-site process to recover P_*i*_ from anaerobic sludge digestion liquor in a small WWTP (**b**)

A 1000-L reactor of 1.3 m height and 1.2 m diameter was made of 1-cm-thick reinforced plastic and set up on a 1.5-tonne motor truck using a steel frame (Fig. 1a). The lower part of the reactor, which had the shape of an inverted circular cone, was designed to collect P_*i*_-rich solids by free sedimentation. The reactor was equipped with an agitator having two stirring blades for mechanical mixing. One inlet valve, which was fitted on the top of the reactor, was connected with an accordion hose for inlet water. Another end of the accordion hose was connected to the outlet of a membrane-type solid–liquid separator for digested sludge in a full-scale WWTP (Fig. 1b).

Two outlet valves were fitted on the side of the reactor to drain the supernatant after the free sedimentation of P_*i*_-rich solids. P_*i*_-rich solids were withdrawn from the bottom of the reactor through an outlet valve. P_*i*_-rich solids were filtered using a self-made filter system on which no mechanical pressure was applied. To prepare the filter system, a 25-cm-diameter filter cloth tube (Public Sheet #200, Asahi Kasei Geotechnologies Co., Tokyo, Japan) was tied at one end with a flexible plastic band and hooked on a steel pipe frame. Since the filter cloth tube is widely used for civil engineering work, it is tough, easy to handle, and available at a cheap price. A 250-L plastic container was placed under the steel pipe frame to collect the filtrate. This filtration system was set up on the ground beside the motor truck before being used.

On-site P_*i*_ recovery experiments were performed using the mobile plant at a full-scale WWTP located in the Osaka area of Japan. This WWTP employs a Bio-P process (alternative anaerobic and aerobic activated sludge process) to remove P_*i*_ from wastewater. P_*i*_-rich sludge is subjected to anaerobic digestion after being concentrated by centrifugation. After measuring the initial P_*i*_ concentration, 8.1–9.8 L of A-CSHs or acid-treated CS slurry (590–720 g dry weight) was added to the reactor in order to set the Ca/P molar ratio at 2.0. After 20 min of mixing, P_*i*_-rich solids were allowed to settle for 30 min. Then, approximately 830 L of the supernatant was drained from the reactor by operating the two outlet valves fitted on the side of the reactor (Fig. 1b). The remaining 170 L of water was withdrawn from the bottom of the reactor and poured into filter cloth bags. After 90 min of filtration, the filter cake was removed from the bags and taken to a laboratory for chemical analyses.

### Plant cultivation test

Andosol, a dark brown soil originally formed from volcanic materials (Odongo et al. 2007), was used for pot testing. Before potting, the soil was air dried and sieved (< 2 mm). Approximately 410 g soil was hand-packed into a plastic pot of 6.5 cm depth and 11.3 cm diameter. All pots were watered to field capacity. The efficacy of recovered P product as a P_*i*_ fertilizer was compared with those of two commercial P_*i*_ fertilizers, Gifu-no-daichi (JA Gifu, Japan) and calcium superphosphate (Kureha Co., Tokyo, Japan). The recovered P product and Gifu-no-daichi were applied to the plant pots at doses of 100, 200, 300, and 400 mg of C-P_2_O_5_ per pot. Calcium superphosphate was applied to the plant pots at a dose of 100 mg of C-P_2_O_5_ per pot. Fifty mg of nitrogen and 42 mg of potassium were applied as ammonium sulfate and potassium chloride to all pots, respectively. Control experiments were conducted by plant pots without the use of fertilizer. To start the plant growth tests, 20 seeds of the leaf vegetable Komatsuna (*Brassica rapa* L. var. *perviridis*) were sown on the soil surface. After 3, 5, and 7 days of cultivation, the germination rate was estimated by the naked eye. Leaf length was measured with a ruler after 7, 14, and 22 days of cultivation. Then, all crops were harvested after 22 days of cultivation to measure their live weight.

## Results

### Characterization of P_*i*_ adsorbents

**Fig. 2:**
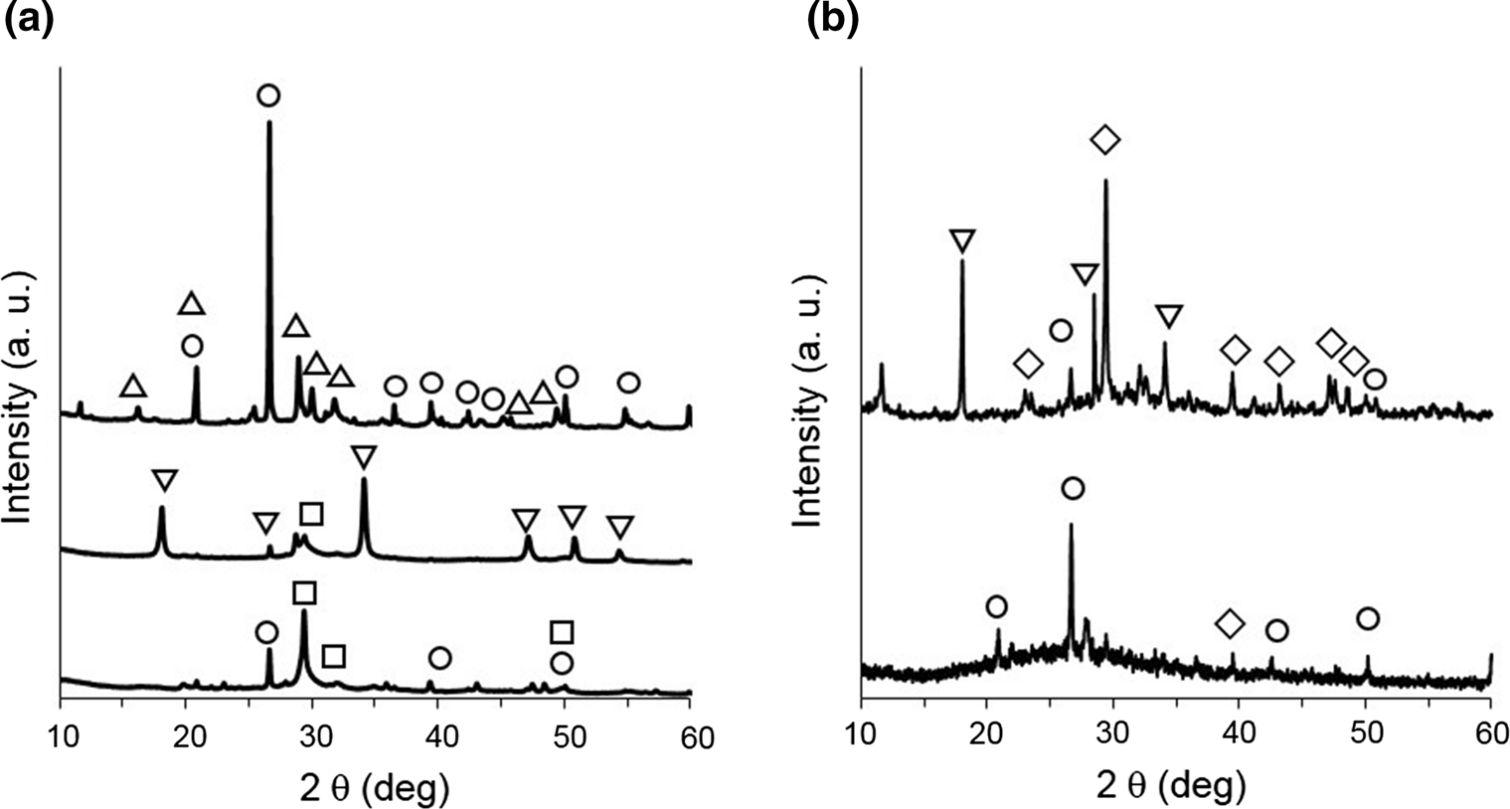
Powder X-ray diffraction patterns. **a** ALC particles (top) and chemically synthesized A-CSHs before (middle) and after (bottom) being washed with distilled water. **b** CS before (top) and after (bottom) acid treatment. Symbols: quartz (circles); tobermorite (triangles); Ca(OH)_2_ (inverted triangles); semicrystalline CSHs (squares); CaCO_3_ (diamonds)

The chemically synthesized A-CSHs had a Ca/Si molar ratio of approximately 2.0–3.5. This was about 4.1–7.1 times greater than that of ALC (Ca/Si = 0.49). The mean particle size of the chemically synthesized A-CSHs was 19 μm, which was less than that of ALC particles (21 μm). Dried A-CSHs had an average surface area of 48 m^2^ g^−1^, approximately 1.3–1.8 times greater than that of ALC particles (38 m^2^ g^−1^). Transmission electron microscopy showed thin platy-layer structures in chemically synthesized A-CSHs and ALC particles. XRD analysis showed that ALC particles exhibited several peaks corresponding to tobermorite and quartz, while dried A-CSHs exhibited several peaks characteristic of Ca(OH)_2_ (Fig. 2a). A-CSHs showed no peaks corresponding to tobermorite, regardless of whether they were washed with distilled water. Small peaks corresponding to semicrystalline CSHs were observed with A-CSHs at approximately 0.304 nm (corresponding to 29.4° 2*θ*). After removing free Ca(OH)_2_ by washing, these peaks became more visible at 0.304, 0.280, and 0.182 nm (corresponding to 29.4, 32.0, and 50.1° 2*θ*, respectively). This semicrystalline structure was likely formed during drying A-CSHs at 100 °C for 10 h (Houston et al. 2009).

The main components of CS particles were SiO_2_ (19.3 mass%) and CaO (41.0 mass%), indicating that the Ca/Si molar ratio was 2.28. The CS particles also contained significant amounts of Al_2_O_3_ and Fe_2_O_3_ (6.3 and 2.3 mass%, respectively). However, no significant amounts of heavy metals such as As, Cd, Pb, Ni, and Cr were detected in CS particles. XRD analysis showed that CS particles exhibited several peaks corresponding to quartz, Ca(OH)_2_, and CaCO_3_ (Fig. 2b). None of the other peaks were clearly assigned because of the complexity and low crystallinity of hydrated cement (Iizuka et al. 2012). Acid-treated CS showed several peaks corresponding to quartz. However, no significant peak was detected for Ca(OH)_2_ and CaCO_3_. Acid-treated CS showed a broad peak in the 2*θ* range of 15–40°, suggesting that the amorphous structure of the CS particles became more prevalent after the acid treatment. This was also suggested by the SEM images of CS particles before and after the acid treatment (data not shown).

### P_*i*_ recovery potential

**Fig. 3:**
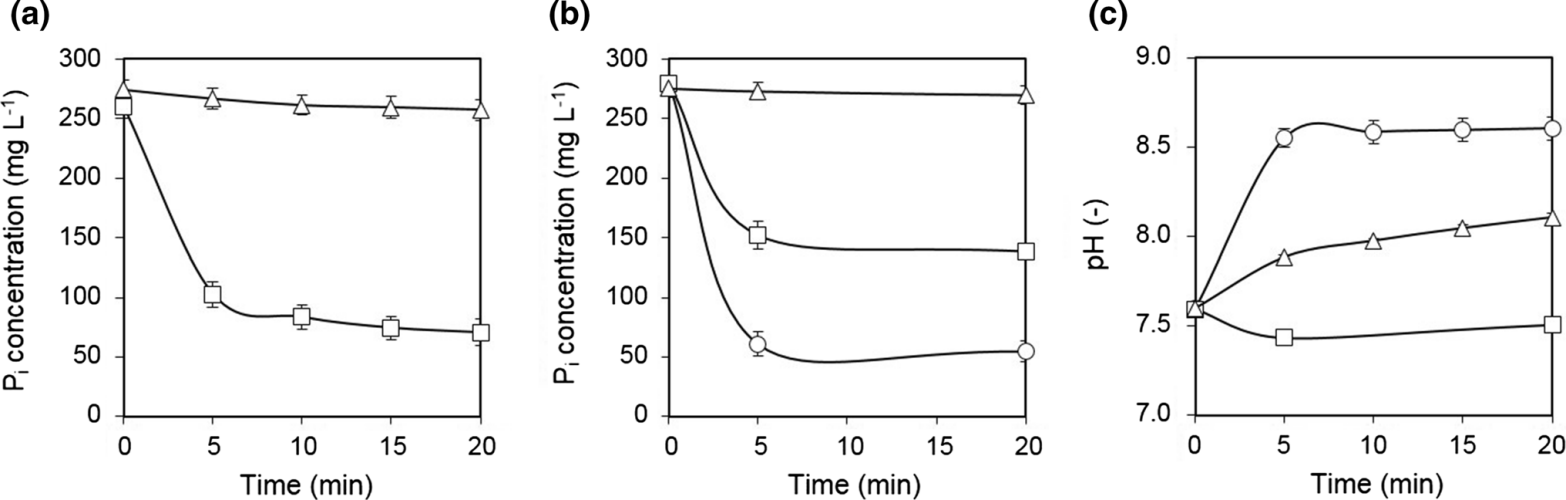
Time-course data on P_*i*_ removal and pH from test solution. **a** P_*i*_ removal by chemically synthesized A-CSHs (squares) and ALC particles (triangles). **b** P_*i*_ removal by untreated CS (triangles), acid-treated CS (squares), and acid-treated CS at an optimum pH 8.5 (circles). **c** pH changes of the reaction mixture in the course of P_*i*_ removal with A-CSHs (circles), ALC particles (triangles), and acid-treated CS (squares). Data points represent the means ± standard deviations for three independent experiments

P_*i*_ removal was first examined in laboratory by adding 1.5 g L^−1^ of chemically synthesized A-CSHs to test solution at a Ca/P molar ratio of 3.5. Preliminary experiments had shown that this molar ratio was optimal for P_*i*_ removal by A-CSHs. The chemically synthesized A-CSHs removed 60% P_*i*_ from the test solution after only 5 min of mixing (Fig. 3a). When the dosage of A-CSHs was increased up to 3.0 g L^−1^, nearly all P_*i*_ was removed from the test solution at 5 min (data not shown). For comparison, ALC particles were added to the test solution at the same concentration of 1.5 g L^−1^. However, ALC particles removed only 8% P_*i*_ from the test solution at 20 min (Fig. 3a). When the concentration of ALC particles was increased tenfold to 15 g L^−1^, about 80% P_*i*_ was removed from the test solution at 20 min (data not shown). In this respect, it must be noted that increasing the dosage of adsorbents unavoidably decreases the P_*i*_ content of recovered product on a dry weight basis, thereby lowering its fertilizer value.

The ability of CS to remove P_*i*_ from aqueous solution was remarkably enhanced by the acid treatment (Fig. 3b). When 0.5 g untreated CS particles were added to the test solution at a Ca/P molar ratio of 2.5 (the optimum molar ratio for acid-treated CS), they removed only 3% P_*i*_ in 60 min. In contrast, acid-treated CS slurry removed 45% P_*i*_ from the test solution in 5 min at the same Ca/P molar ratio. Interestingly, while chemically synthesized A-CSHs and ALC slightly increased pH in the course of P_*i*_ removal, no significant pH increase was detected with acid-treated CS (Fig. 3c). Since nearly all Ca(OH)_2_, which existed in CS, was dissolved by the HCl-soaking treatment (see Fig. 2b), no significant release of Ca^2+^ occurred from the solid fraction of acid-treated CS during P_*i*_ recovery (data not shown). This may explain why acid-treated CS did not increase pH in the course of P_*i*_ recovery. It seems to be of practical importance that high pH is not required for P_*i*_ recovery using acid-treated CS. The P_*i*_ removal efficiency of acid-treated CS could be changed by changing the initial pH of the reaction mixture. The P_*i*_ removal by acid-treated CS slurry increased when the initial pH was increased from 7.6 to 8.5. Acid-treated CS removed 82% P_*i*_ in 5 min at the optimum pH of 8.5 (Fig. 3b). When the liquid and solid fractions of acid-treated CS slurry were separated by centrifugation, the solid fraction showed only 6% P_*i*_ removal from the test solution at 60 min. By contrast, the liquid fraction of acid-treated CS slurry removed 63% P_*i*_ from the test solution (data not shown).

**Fig. 4:**
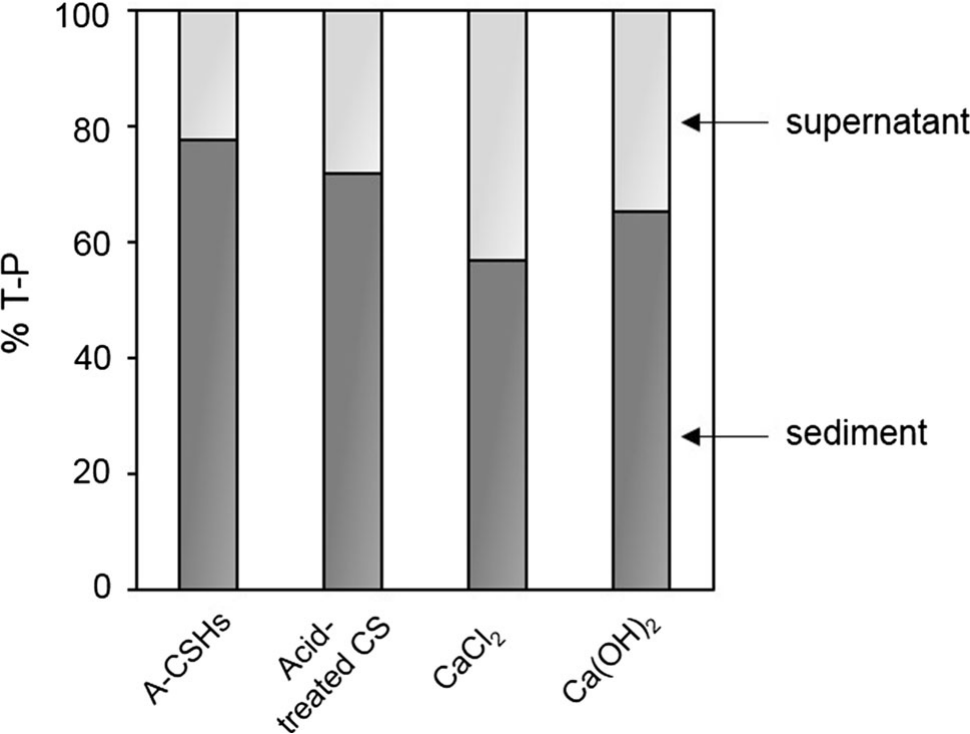
Settleability test of P removed by chemically synthesized A-CSHs, acid-treated CS, CaCl_2_, or Ca(OH)_2_. After 20 min of P_*i*_ removal, 200 mL reaction mixture was subjected to 10 min of free sedimentation. About 180 mL (90% of the original volume) of the supernatant was gently removed using a siphon. Settleability was evaluated by determining the percentage of total P that remained in the cylinder (dark gray) after siphoning the supernatant

Chemically synthesized A-CSHs showed better P_*i*_ settleability than did CaCl_2_ and Ca(OH)_2_ (Fig. 4). A-CSHs, CaCl_2_, and Ca(OH)_2_ settled 78, 57, and 65% of the total P from test solution, respectively, after 10 min of free sedimentation. On the other hand, acid-treated CS could precipitate 72% P_*i*_ in 5 min. Interestingly, the liquid fraction of acid-treated CS slurry showed lower P_*i*_ settleability than did uncentrifuged acid-treated CS slurry (data not shown). Uncentrifuged acid-treated CS slurry precipitated approximately 72% P_*i*_ in 5 min of free sedimentation, while only 48% P_*i*_ was settled by the liquid fraction alone. Moreover, P_*i*_ precipitation with CaCl_2_ was significantly enhanced by the addition of the solid fraction of acid-treated CS slurry. The enhancement of P_*i*_ settleability by the solid fraction was significantly reduced after treatment with 1 M NaCl (data not shown).

### On-site P_*i*_ recovery test

**Fig. 5:**
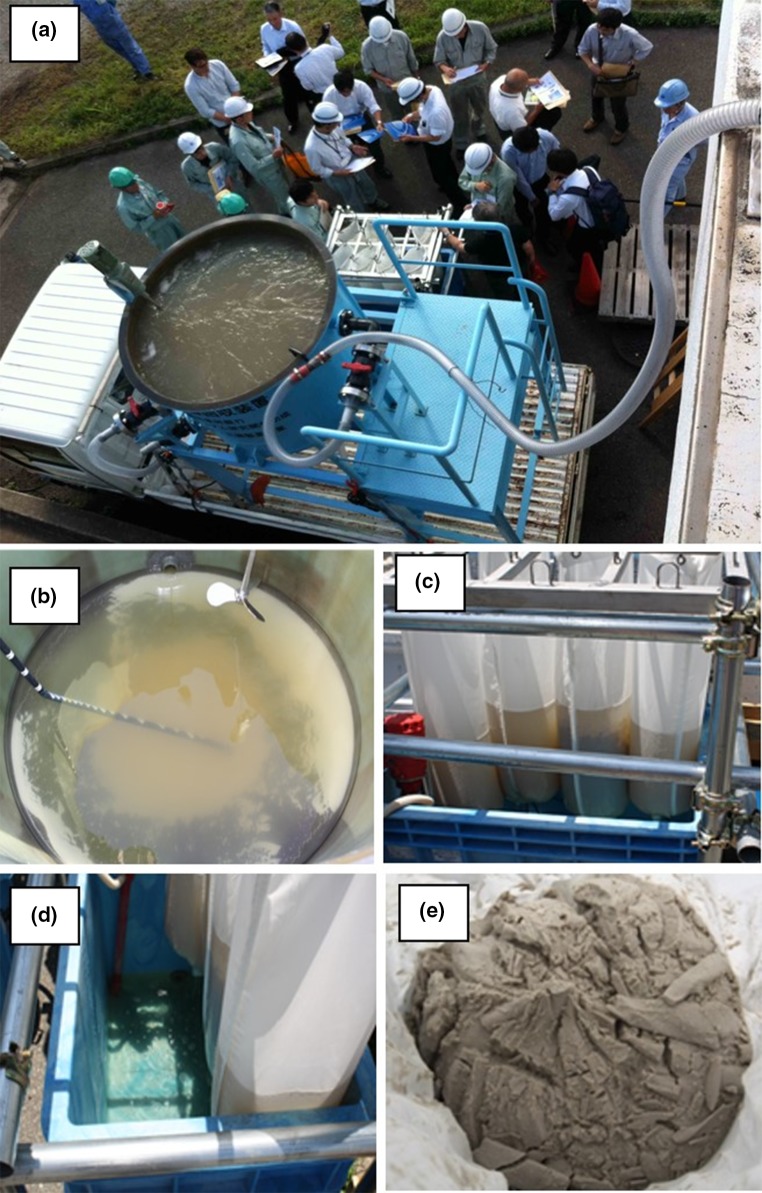
On-site P_*i*_ recovery experiment using a mobile plant. Photographs show the mobile plant (**a**), the P_*i*_-rich solids at the reactor bottom after free sedimentation (**b**), the filter cloth bags to recover P_*i*_-rich solids (**c**), the filtrate from the filter cloth bags (**d**), and the recovered P product (**e**)

On-site P_*i*_ recovery tests were carried out at a full-scale WWTP (Fig. 5a) using chemically synthesized A-CSHs and acid-treated CS. The mobile plant was used to recover P_*i*_ from anaerobic sludge digestion liquor (rejected water from a membrane-type solid–liquid separator for digested sludge) at the WWTP. In a preliminary run, it was observed that large sludge flocs were present in the anaerobic sludge digestion liquor because of the backwash of the solid–liquid separation membrane for digested sludge. Large sludge flocs unavoidably contaminated recovered P product and decreased the C-P_2_O_5_ content on a dry weight basis. To reduce the contamination, anaerobic sludge digestion liquor was passed through a self-made cotton filter before feeding into the reactor (Fig. 1b). The pH of the anaerobic sludge digestion liquor was nearly constant ranging from 7.7 to 8.1, while the P_*i*_ concentration varied from 168 to 202 mg P_*i*_ L^−1^.

**Fig. 6:**
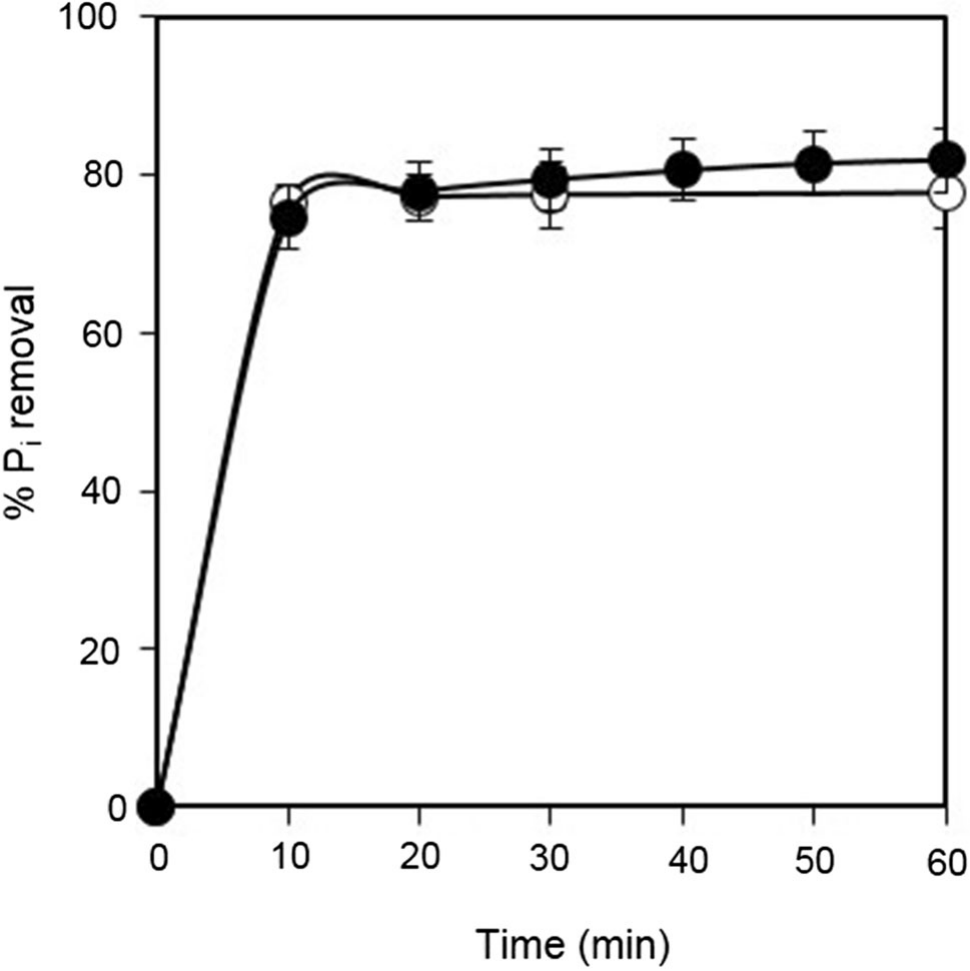
Time-course data on the % P_*i*_ removal by chemically synthesized A-CSHs (open circles) and acid-treated CS (closed circles) in on-site P_*i*_ recovery experiments. Data points represent the means ± standard deviations for three independent experiments

The P_*i*_ recovery experiment was started by adding either chemically synthesized A-CSHs or acid-treated CS slurry to the reactor at a Ca/P molar ratio of 2.0. The Ca/P molar ratio was somewhat smaller than that employed in laboratory experiments (i.e., 2.5–3.5). This was required for reducing excess Ca^2+^ which may leave from the reactor without being used for P_*i*_ removal. Soon after the start of the mechanical mixing, both chemically synthesized A-CSHs and acid-treated CS showed high rates of P_*i*_ removal (Fig. 6). They removed approximately 80% P_*i*_ from the anaerobic sludge digestion liquor after 5 min. Then P_*i*_ removal gradually increased to 82% by 60 min. The pH of the reaction mixture slightly increased to 8.4–8.8 after 60 min (data not shown).

After 30 min of free sedimentation, about 830 L of the supernatant was drained from the reactor (Fig. 5b). Sediments were easily poured into filter cloth bags (Fig. 5c). Then the filtration was carried out without mechanical pressure being applied. As shown in Fig. 5d, the filtrate in the plastic container was clear, suggesting that P_*i*_-rich solids were effectively recovered by the filter cloth bags. After 90 min of filtration, the filter cake was removed from the bags for chemical analyses (Fig. 5e). Mass balance calculation showed that 72–85% P_*i*_ was recovered from the anaerobic sludge digestion liquor, while 13–23% P_*i*_ was lost in the drainage water from the reactor. The loss of P_*i*_ due to filtration was only 2–5%. There was no significant difference in the P_*i*_ recovery efficiency between chemically synthesized A-CSHs and acid-treated CS. Despite the high T-CO_2_ of anaerobic sludge digestion liquor (1280 mg L^−1^), no carbonate inhibition was observed with P_*i*_ removal in the on-site experiments.

### P_*i*_ recovery products

No significant difference was observed with the P products recovered by chemically synthesized A-CSHs and acid-treated CS. The recovered P product contained 83–87% moisture before drying for chemical analyses. The ignition loss of the product ranged from 19 to 23%. After drying at 105 °C for 24 h, the product contained 19–24% of the dry weight as P_2_O_5_. The P_2_O_5_ content was nearly identical to the C-P_2_O_5_ content (18–24%). On average, total potassium (K_2_O), total nitrogen (T-N), and CaO contents were 0.1, 0.5, and 38%, respectively, on a dry weight basis. Cd was never detected in the recovered products. The contents of As, Pb, Ni, and Cr were less than 4.0, 4.0, 2.0, and 12.0 mg kg^−1^, respectively. These values were much lower than their regulatory levels (840, 100, 300, and 500 mg kg^−1^, respectively) for fertilizer in Japan. When the recovered P product was applied to andosol at a dose of 100 mg C-P_2_O_5_ per pot, the germination rate of Komatsuna (*Brassica rapa* L. var. *perviridis*) seeds was higher than 90% after 5 days (data not shown). There was no significant difference in the germination rate between the recovered P product and the commercial P_*i*_ fertilizers (Gifu-no-daichi and superphosphate). Increasing the dose of the recovered P product had no significant effect on the germination rate. The live weight of Komatsuna was 9.1, 9.5, and 7.8 g per pot at 22 days after applying 100 mg P_2_O_5_ per pot of the recovered P product, Gifu-no-daichi, and superphosphate, respectively. Komatsuna took up approximately 3.5–4.5 mg P at 22 days, accounting for approximately 8–10% of applied P. No significant plant growth was observed without the addition to fertilizer to andosol. The growth of Komatsuna was enhanced by increasing the dose of the recovered P product. No abnormal plant growth was observed with Komatsuna at the dose of 400 mg C-P_2_O_5_ per pot.

## Discussion

Chemically synthesized A-CSHs had an average surface area of 48 m^2^ g^−1^, approximately 1.3–1.8 times greater than that of ALC particles (38 m^2^ g^−1^). While A-CSHs removed 60% P_*i*_ from the test solution after 5 min of mixing, ACL particles showed only 8% P_*i*_ removal at 20 min (Fig. 3a). Obviously, the high ability of A-CSHs to remove P_*i*_ cannot be explained simply by their relatively large surface area. Previously, we have shown that chemically synthesized A-CSHs consisted of silicate polymers that are linked to each other through ion binding with Ca^2+^ using ^29^Si MAS-NMR analysis (Okano et al. 2015). This structure of A-CSHs was distinctly different from that of tobermorite (Shaw et al. 2000). Tobermorite, which is a principal component of ALC particles (Liu et al. 2001), consists of CaO polyhedral sheets sandwiched between single silicate chains (Shaw et al. 2000). These composite layers have three types of linkages: Si–O–Si, Si–O–Ca, and Si–OH. The poor P removability of ALC particles is likely due to the poor reactivity of these linkages. Unfortunately, ^29^Si MAS-NMR analysis failed to show the detailed structure of acid-treated CS because of its high complexity.

Chemically synthesized A-CSHs could readily release Ca^2+^, when they were dispersed in test solution (data not shown). The initial steps of nucleation of Ca–P_*i*_ in aqueous solution have been speculated using quantum/classical molecular mechanics simulation (Zahn 2004). The molecular mechanics simulation has revealed that a [Ca^2+^–(HPO_4_)^2−^–Ca^2+^]^2+^ aggregate can be formed at the initial stage of nucleation of Ca–P_*i*_ in aqueous solution. Since A-CSHs can acquire negative electrical charges after releasing Ca^2+^, it seems possible that the [Ca^2+^–(HPO_4_)^2−^–Ca^2+^]^2+^ ion triple ionically binds to the negatively charged A-CSHs, forming Ca-P_*i*_–silicate ion aggregates. On the other hand, P_*i*_ precipitation with CaCl_2_ was significantly enhanced by the addition of the solid fraction of acid-treated CS slurry. However, the enhancement of P_*i*_ settleability by the solid fraction was significantly reduced after treatment with 1 M NaCl. These results suggested that the surface electric charge of particles in acid-treated CS slurry was also responsible for forming ion aggregates, thereby enhancing P_*i*_ settleability.

Recyclable calcium silicates are available almost free of charge from the cement and steel industry. About 197 million tons (Mt) of fresh concrete is produced annually in Japan, and about 1–2% of fresh concrete prepared for construction is discarded as concrete sludge (Tsunashima et al. 2012). On the other hand, about 15 Mt of steelmaking slag is annually produced in the iron and steelmaking industry in Japan. The valorization of recyclable CSHs is critical to the resource efficiency in the cement and steel industry. Steel slag has been used as a fertilizing material containing Ca, Si, Mg, Mn, Fe, and P in China and Japan. Slag-based silicon fertilizers have beneficial effects on the growth and disease resistance of rice (Ning et al. 2014). Actually, Japan has long experience regarding the safe use of slag-based fertilizer in agriculture. The Japanese Fertilizer Regulation Act specifies the upper limit of heavy metals, including Ni, Cr, and Ti, for slag-based fertilizer. From nutritional perspectives, Co, Cr, Cu, Mo, Mn, Se, and Zn are micronutrients required for plant growth. V is also considered as a substitute for Mo in plant physiology. There are many types of steel slag produced during the steelmaking process. Since their chemical composition considerably differs, it is possible to select slag that is most suited for target fertilizer.

Although many previous workers have reported the use of crystalline-type CSHs as a P_*i*_ adsorbent or a seed for hydroxyapatite crystallization (Berg et al. 2005; Chen et al. 2009), no attempt has been made to enhance the ability of calcium silicates to remove P_*i*_ by soaking them in hydrochloric acid. The present technology has the potential to expand the usefulness of recyclable calcium silicates which are abundantly available from the cement and steel industries. The cost related to producing acid-treated CS is comparable to that of disposal by landfill, because the disposal of CS by landfill also needs solid–liquid separation followed by neutralization with a strong acid. Rather, if acid-treated CSHs could be used as a substitute for lime, this would significantly improve the resource efficiency of the cement and steel industries.

Like chemically synthesized A-CSHs, acid-treated CS could readily form insoluble ion aggregates with P_*i*_, thereby enabling high P_*i*_ settleability without adding any other chemical coagulants. P_*i*_-rich solids could be recovered by filtration using inexpensive filter cloth bags without mechanical pressure being applied. The recovered P product had a high C-P_2_O_5_ content (around 18% on a dry weight basis). This value was higher than the minimum requirement for C-P_2_O_5_ (15% on a dry weight basis) in byproduct P_*i*_ fertilizer in Japan. The product with a C-P_2_O_5_ content of 15% or higher could never have been obtained by untreated CS or ALC particles. On the contrary, levels of toxic heavy metals such as Cd, As, Pb, Ni, and Cr were much lower than their regulation standards for fertilizer in Japan. Since the content of water-soluble P_2_O_5_ in the recovered P product was less than 0.01%, the recovered product could be used as a slow-release P_*i*_ fertilizer.

Land application of sewage sludge is practically prohibited in some countries in Europe as well as Japan. For these countries, it is imperative to recover P_*i*_ from sewage sludge in order to locally recycle P_*i*_ from wastewater to farmland. In this respect, Japan has long experience in commercial operation of full-scale plants to recover P_*i*_ at large-scale WWTPs (Ohtake and Okano 2015). However, since these technologies cost much money for plant construction and operation, they are not suited for P_*i*_ recovery in small WWTPs. In Japan, plants having the sewage treatment capacity smaller than 25 000 t d^−1^ (approximately 100 000 p.e.) account for about 90% of operating sewage treatment plants. However, they treat only 25% of a total of c. 40 000 000 m^3^ of sewage per day. Since the P concentration is comparable, it is likely that smaller sewage works in Japan have the potential to recover approximately 25% of P in sewage (c. 13 000 t P a^−1^). In addition to 2100 sewage treatment plants, about 1000 blackwater treatment plants are operating in Japan. Blackwater treatment plants are all small, having the capacity less than 100 m^3^ of blackwater per day. However, since the T-P concentration of human excreta is typically 200–300 mg P L^−1^ (c. 40–60 times higher than that of sewage), blackwater treatment plants have the P recovery potential of c. 2300 t P a^−1^. Up to now, P recovery has been implemented in c. 10 blackwater treatment plants, contributing to P recycling in rural areas.

In the present study, on-site P_*i*_ recovery experiments were performed at a sewage treatment plant operating in a Bio-P mode. If P_*i*_ is chemically precipitated with Al^3+^ or Fe^3+^, P_*i*_ remains in sludge even after anaerobic digestion. This is unfavorable for P_*i*_ recovery from sludge slurry. However, many small plants are operated without nutrient removal in rural areas. Dehydrated sludge is often collected by truck and transported to a remote sludge treatment center. It seems likely that P_*i*_ could be recovered from anaerobic sludge digestion liquor or incinerated sludge ash at a remote sludge treatment center. In addition, there is potential for P recovery in approximately 1000 blackwater treatment plants. The government offers subsidies to P recovery at blackwater treatment plants to promote P recycling in rural areas. The mobile plant used in the present study is easily conveyed by a small motor truck and readily installed in a space smaller than 15 m^2^. Therefore, it seems likely to have the potential to offer a simple, compact service for on-site P recovery in these small plants. The recovered product could be used as a source of P_*i*_ for recycled fertilizer in local areas. For example, the recovered P product can be added to wood waste compost, thereby increasing its fertilizer value. The P_*i*_-amended wood waste compost may be applied to farmland near to a small plant as a recycled organic fertilizer. This could help the local recycling of P in rural areas.

The amorphous CSHs-based technology for P_*i*_ recovery is in an early phase of development. Much work is needed for the automatic control and total cost estimation of P_*i*_ recovery process as well as the fertilizer efficacy and safety evaluation of recovered P products. It may also need to address the quality variation of recyclable calcium silicates, since this can unavoidably affect the performance of bifunctional adsorption–aggregation agents on P_*i*_ recovery. However, if these issues are properly addressed, the present technology could offer a simple, compact option to P recycling from wastewater to farmland in rural areas.

## Conclusions

A simple technology for P_*i*_ recovery has been developed using a bifunctional adsorption–aggregation agent. The bifunctional agent was prepared simply by soaking recyclable calcium silicates in hydrochloric acid solution. On-site experiments using a mobile plant showed that approximately 80% P_*i*_ could be recovered from anaerobic sludge digestion liquor at a wastewater treatment plant. This technology has the potential to offer new technology options to local small plants for recycling P from wastewater to farmland in rural areas.
